# Supplementary material for: Ferroptosis-Related Molecular Clusters and Diagnostic Model in Rheumatoid Arthritis
Source: Int J Mol Sci. 2023 Apr 16;24(8):7342. doi: 10.3390/ijms24087342 (PMC10138921; doi:10.3390/ijms24087342)
Supplement: Supplementary file 1 [file ijms-24-07342-s001.zip › ijms-2264761-supplementary.pdf]

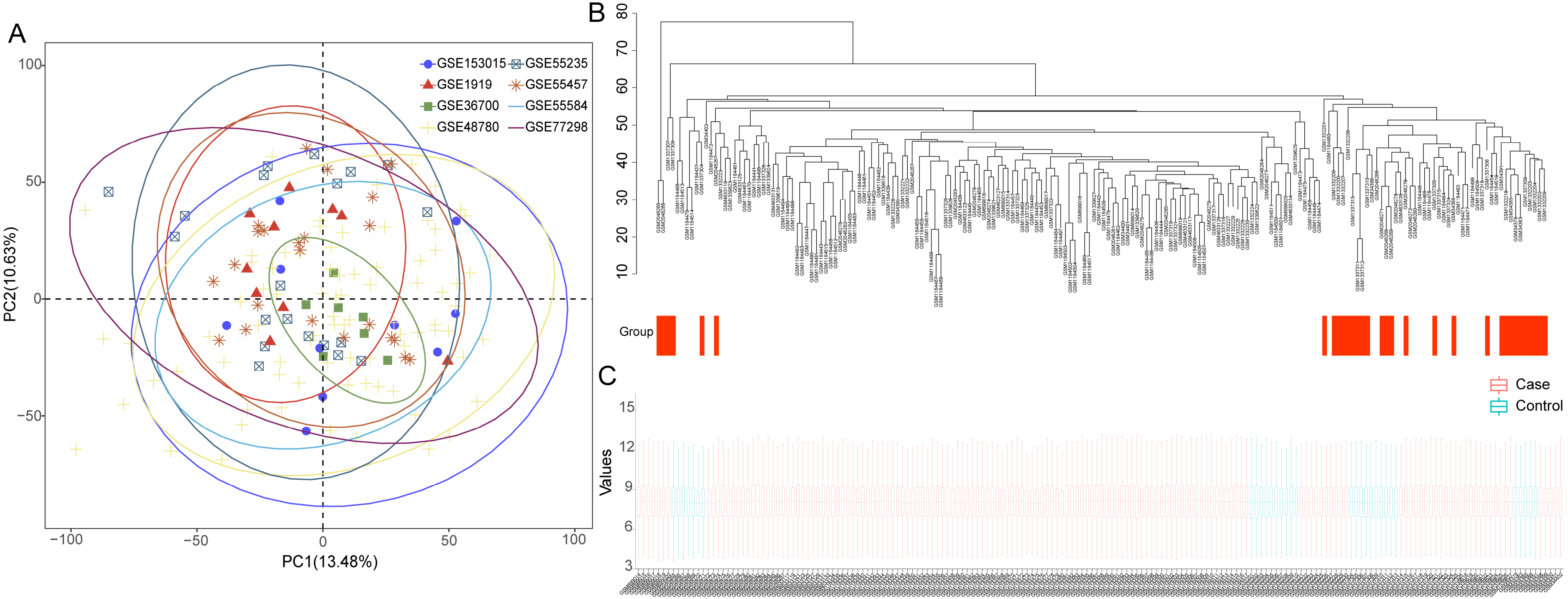

Figure S1. Results of data preprocessing evaluations. (A) PCA maps between batches of different data sets were removed, which revealed that the different data sets were mixed together without significant separation. (B) Sample clustering results; red represents HC samples and white represents RA samples. (C) Boxplot evaluation of different sample data.
